# Identification of differentially expressed genes in cutaneous squamous cell carcinoma by microarray expression profiling

**DOI:** 10.1186/1476-4598-5-30

**Published:** 2006-08-08

**Authors:** Ingo Nindl, Chantip Dang, Tobias Forschner, Ralf J Kuban, Thomas Meyer, Wolfram Sterry, Eggert Stockfleth

**Affiliations:** 1 Department of Dermatology, Charité, Skin Cancer Center Charité, University Hospital of Berlin, Charitéplatz 1, D-10117 Berlin, Germany; 2 Institute of Biochemistry, Charité, University Hospital of Berlin, Monbijoustr. 2, D-10098 Berlin, Germany; 3 Institut of Pathology and Molecularbiology (IPM), Lademannbogen 61, D-22339 Hamburg, Germany

## Abstract

**Background:**

Carcinogenesis is a multi-step process indicated by several genes up- or down-regulated during tumor progression. This study examined and identified differentially expressed genes in cutaneous squamous cell carcinoma (SCC).

**Results:**

Three different biopsies of 5 immunosuppressed organ-transplanted recipients each normal skin (all were pooled), actinic keratosis (AK) (two were pooled), and invasive SCC and additionally 5 normal skin tissues from immunocompetent patients were analyzed. Thus, total RNA of 15 specimens were used for hybridization with Affymetrix HG-U133A microarray technology containing 22,283 genes. Data analyses were performed by prediction analysis of microarrays using nearest shrunken centroids with the threshold 3.5 and ANOVA analysis was independently performed in order to identify differentially expressed genes (p < 0.05). Verification of 13 up- or down-regulated genes was performed by quantitative real-time reverse transcription (RT)-PCR and genes were additionally confirmed by sequencing. Broad coherent patterns in normal skin *vs*. AK and SCC were observed for 118 genes.

**Conclusion:**

The majority of identified differentially expressed genes in cutaneous SCC were previously not described.

## Background

Nonmelanoma skin cancer (NMSC), encompassing both basal cell carcinoma (BCC) and squamous cell carcinoma (SCC), is the most common cancer in Caucasians, and over the last decade incidence has been increased dramatically worldwide [1]. Actinic keratosis (AK) is an early stage of SCC and approximately 10% of cases progress to SCC [2-4]. Ultraviolet radiation (UV) is the major risk factor for this disease [5,6]. Carcinogenesis is a multi-step process indicated by a series of genes that are up- or down-regulated during tumor progression. In normal vs. cancerous colon tissue only 1–1.5% of approximately 30,000 – 50,000 functional genes per cell were differentially expressed resulting in 548 genes [7]. A comparable quantity was identified in breast cancer cells exhibiting 700 dysregulated genes [8]. Thus, the number of differentially expressed genes seem to be similar in different cancers. Many genes showing increased expression elevated in colon-cancer represent proteins that are considered to be involved in growth and proliferation while there were often attributed to differentiation in normal tissue. Analyzing skin- or head and neck-cell lines, genes that are associated with extracellular matrix production and apoptosis were disrupted in preneoplastic cells during SCC development, whereas genes that are involved in DNA repair or epidermal growth factors were altered at later stages [9]. Transformation of keratinocytes in response to UV-radiation was examined by microarray technology using normal human epidermal keratinocytes and SCC cell lines [10]. This study detected four clusters of differentially expressed genes in normal keratinocytes *vs*. skin cancer cells, which may play a role in the carcinogenic pathway. However, cell lines are often different from human tissues that has been demonstrated for both colon cancer [11] and ovarian cancer [12,13]. Thus, human cancer tissues are superior compared with cell lines analyzing differentially expressed genes. So far, only one study examined differentially expressed genes in NMSC tissue using nylon-filter DNA microarrays analyzing approximately 7,400 genes [14].

In the present study, we evaluated the different expression profile of 22,283 genes in normal skin biopsies *vs*. AK *vs*. cutaneous SCC. We focused on dysregulated genes best characterizing normal skin and NMSC (comprising both AK and SCC). Overall, 42 genes were up-regulated and 76 genes were down-regulated in skin cancer and the majority of differentially expressed genes were not described earlier.

## Results

### Relative expression in normal skin and NMSC

Of the 22,283 transcripts and expressed sequence tags (EST) investigated on each oligonucleotide microarray, 118 genes were detected differentially expressed in normal skin, AK, and SCC by Prediction Analysis of Microarrays (PAM) analysis excluding 81 genes (EST and genes with the description "consensus includes...") (see Methods). For each of the 6 normal skins, 4 AK, and 5 SCC (Table 1), the relative expression of each gene was examined. We have controlled the RNA quality of each human specimen analyzing the fragment sizes, and the ratio of 5'- and 3'-ends using microarray test-chips with 24 human control genes of 6 randomly selected specimens and only non-degraded mRNA specimens were used in this study.

**Table 1:** Data of organ transplant (TX) non-melanoma skin cancer (NMSC) patients or non-TX.

Patient	Sex/age (years)	TX	Time after TX (years)	Normal skin	AK	SCC
1 (BN)	F/60	Kidney	23	Insight lower arm (ILA^a^)	Finger **(1)**^b^	Hand **(1)**
2 (GM)	M/66	Kidney	11	ILA^a^	Lower leg **(2)**	Forehead **(2)**
3 (MP)	M/58	Liver	12	ILA^a^	Lower Leg^c^	Lower Leg **(3)**
4 (DR)	M/73	Heart	9	ILA^a^	Head **(3)**	Forehead **(4)**
5 (JG)	M/69	Kidney	2	ILA^a ^**(1)**	Ear^c^**(4)**	Forehead **(5)**
6 (GH)	M/57	Non-TX		Head **(2)**	NC	NC
7 (WM)	F/61	Non-TX		Cheek **(3)**	NC	NC
8 (WM)	F/61	Non-TX		Head **(4)**	NC	NC
9 (EW)	F/74	Non-TX		Face **(5)**	NC	NC
10 (MG)	M/17	Non-TX		Fundament **(6)**	NC	NC

Normal skin, AK, and SCC biopsies were collected from each TX-patient, and in addition normal skin biopsies were obtained from age-matched immunocompetent patients (non-TX).

^a ^RNA of 5 normal biopsies were pooled.

^b ^The biopsies used for microarray analysis are indicated with numbers in parentheses marked in bold.

^c ^RNA of 2 AK were pooled.

F, female; M, male; AK, actinic keratosis; SCC, squamous cell carcinoma; NC, not collected.

PAM analysis was used to identify genes to classify and to best characterize normal skin, AK or SCC. The rate of misclassification on the basis of individual cross validation plots was 0% (0.0) for normal skin, 25% (0.25) for AK, 20% (0.20) for SCC, and 13% (0.13) for all three classes. The first gene list contained 200 genes best characterizing normal skin (6), AK (4), and SCC (5). Under the exclusion of EST and genes with the description "consensus includes..." (n = 81) we identified 118 dysregulated genes (Table 2).

**Table 2:** Genes identified by Prediction Analysis of Microarrays (PAM) differentially expressed in normal skin compared to non-melanoma skin cancer.

**A) Up-regulated genes (42) in non-melanoma skin cancer.**							**Microarray experiments**			**ANOVA**
								
	**No.**	**Accession No.**	**Symbol**	**Gene**	**Function**	**Localization**	**Change fold T/N**	**Mean of raw signal (normal)**	**Mean of raw signal (AK&SCC)**	**p-value**
**Differentiation**										
	1	AF183421.1	rab22b	Small GTP-bindung protein rab22 (RAB31)	Small GTPase signal transduction.	18p11.3	1.87	6853	12495	n.s.
	2	**NM_006868.1**	RAB31*	RAB31, member RAS oncogene family	Small GTPase signal transduction.	18p11.3	3.30	2637	8437	0.027
**Apoptosis**										
	3	**NM_004834.1**	MAP4K4*	Mitogen-activated protein kinase kinase kinase kinase 4	A member of the serine/threonine protein kinase family, specifically activate MAPK8/JNK.	2q11.2-q12	2.65	1475	3898	0.024
**Proliferation**										
	4	NM_007375.1	TARDBP	TAR DNA binding protein	Transcriptional repressor that binds to chromosomally integrated TAR DNA and represses HIV-1 transcription.	1p36.22	1.48	6779	10441	n.s.
**Metabolism**										
	5	D55674.1	hnRNP D	Heterogeneous nuclear ribonucleoprotein D	Associated with pre-mRNAs in the nucleus and appear to influence pre-mRNA processing and other aspects of mRNA metabolism and transport.	4q21.1-q21.2	1.64	4020	6968	n.s.
	6	**NM_003942.1**	RPS6KA4	Ribosomal protein S6 kinase	RSK (ribosomal S6 kinase) family of serine/threonine kinases	11q11-q13	1.74	3374	6029	0.044
	7	NM_001814.1	CTSC	Cathepsin C (CTSC)	Defects in the encoded protein have been shown to be a cause of Papillon-Lefevre syndrome, an autosomal recessive disorder characterized by palmoplantar keratosis and periodontitis.	11q14.1-q14.3	1.64	11435	18686	n.s.
	8	Z14077.1	YY1	YY1, NF-E1 protein	Transcription factor involved in repressing and activating a diverse number of promoters.	14q	2.08	4185	8939	n.s.
	9	NM_006141.1	DNCLI2	Dynein, cytoplasmic, light intermediate polypeptide 2	Involved in retrograde organelle transport and some aspects of mitosis.	16q22.1	3.23	2225	5692	n.s.
	10	AF061832.1	M4	M4 protein deletion mutant	Appear to influence pre-mRNA processing and other aspects of mRNA metabolism and transport.	19p13.3-p13.2	1.29	11154	14824	n.s.
**Cell communication**										
	11	U65590	IL-1RN*	IL-1 receptor antagonist IL-1Ra	Inhibits the activities of interleukin 1, alpha (IL1A) and interleukin 1, beta (IL1B), and modulates a variety of interleukin 1 related immune and inflammatory responses.	2q14.2	1.75	9599	18079	n.s.
	12	NM_004688.1	NMI*	N-myc (and STAT) interactor	Interacts with NMYC, CMYC, all STATs except STAT2.	2p24.3-q21.3	2.33	2744	6248	n.s.
	13	NM_002416.1	MIG	Monokine induced by gamma interferon	Binding to CXCR3 causes pleiotropic effects, including stimulation of monocytes, natural killer and T-cell migration, and modulation of adhesion molecule expression.	4q21	3.54	1492	10529	n.s.
	14	U72069.1	TNPO1	Karyopherin beta2	Interacts with nuclear localization signals to target nuclear proteins to the nucleus.	5q13.2	2.06	2986	6049	n.s.
	15	BC004489.1	HLA-C	Major histocompatibility complex, class I, C	A central role in the immune system by presenting peptides derived from ER lumen.	6p21.3	1.30	78196	106054	n.s.
	16	L42024.1	HLA-B39	MHC, HLA-B39	A central role in the immune system by presenting peptides derived from ER lumen.	6p21.3	1.35	78040	114101	n.s.
	17	NM_005516.1	HLA-E	Major histocompatibility complex, class I, E	A central role in the immune system by presenting peptides derived from ER lumen.	6p21.3	1.76	24252	43847	n.s.
	18	**NM_006096.1**	NDRG1	N-myc downstream regulated gene 1	Involved in stress responses, hormone responses, cell growth, and differentiation.	8q24.3	2.60	17233	44914	0.024
	19	**AF313468.1**	lectin-1	Dendritic cell-associated C-type lectin-1	Diverse functions, such as cell adhesion, cell-cell signalling, glycoprotein turnover, and roles in inflammation and immune response.	12p12.3-p13.2	3.61	1163	4146	0.019
	20	**U88964**	HEM45	HEM45	HIV-1 Tat upregulates the interferon-responsive gene expression of HEM45.	15q26	3.24	982	3293	0.019
	21	**NM_000418.1**	IL4R*	Interleukin 4 receptor	Development of allergic reactions and have been shown to modulate the function of monocytes and macrophages.	16p11.2-12.1	2.41	2519	6159	0.014
	22	**NM_015986.1**	CREME9	Cytokine receptor-like molecule 9	Thought to be involved in signal transduction.	17q11.2	1.58	4335	6950	0.015
	23	**NM_002087.1**	GRN*	Granulin	A role in the development of prostatic intraepithelial neoplasia.	17q21.32	1.77	14453	25978	0.034
**Adhesion**										
	24	**AK023406.1**	FLJ13344	FLJ13344 fis	High homology to the actin and microtubules binding protein ABP620.	1p32-p31	2.49	3656	9107	0.038
	25	U03271	CAPZB	F-actin capping protein beta subunit	Regulates growth of the actin filament by capping the barbed end of growing actin filaments.	1p36.1	1.35	11809	16060	n.s.
	26	NM_005572.1	LMNA	Lamin AC	Lamin proteins are thought to be involved in nuclear stability, chromatin structure and gene expression.	1q21.2-q21.3	1.47	13531	19844	n.s.
	27	**AB010427.2**	WDR1	NORI-1	May help induce the disassembly of actin filaments.	4p16.1	2.11	7418	15927	<0.001
	28	**NM_004893.1**	H2AFY	H2A histone family, member Y	A member of the histone H2A family.	5q31.3-q32	1.39	18975	26758	0.048
	29	**NM_001101.2**	ACTB	Actin, beta	Conserved proteins that are involved in cell motility, structure and integrity.	7p15-p12	1.44	120864	178342	0.026
	30	**NM_000700.1**	ANXA1	Annexin A1	Located on the cytosolic face of the plasma membrane.	9q12-q21.2	1.74	35910	64023	0.043
	31	**NM_002160.1**	TNC*	Hexabrachion (tenascin C, cytotactin) (HXB),	Spliced tenascin-C has important roles in tumor progression of breast cancer.	9q33	4.60	1819	8694	0.038
	32	BF338947	1–8U	Interferon induced transmembrane protein 3	Transmembrane protein.	11p15.5	1.70	20588	33223	n.s.
	33	**NM_002421.2**	MMP1*	Matrix metalloproteinase 1	Involved in the breakdown of extracellular matrix in normal physiological processes (embryonic development, reproduction, and tissue remodeling), in disease processes (arthritis, metastasis).	11q22.3	> 10	293	18006	0.024
	34	**J00269.1**	KRT6A	Human 56 k cytoskeletal type II keratin	Member of the keratin gene family.	12q12-q13	3.79	42604	161154	0.026
	35	**NM_006825.1**	CKAP4	Transmembrane protein	Required for cell adhesion.	12q24.11	1.68	12780	22154	0.023
	36	NM_005561.2	LAMP1	Lysosomal-associated membrane protein 1	Possible a role in tumor cell metastasis.	13q34	1.28	25495	33858	n.s.
	37	**NM_001793.1**	CDH3	Cadherin 3, type 1, P-cadherin (placental)	Calcium-dependent cell-cell adhesion glycoprotein, mutations in this gene have been associated with congential hypotrichosis with juvenile macular dystrophy.	16q22.1	3.63	3819	15934	0.043
	38	NM_004360.1	CDH1*	Cadherin 1, type 1, E-cadherin (epithelial)	Calcium dependent cell-cell adhesion glycoprotein, mutations in this gene are correlated with gastric, breast, colorectal, thyroid and ovarian cancer.	16q22.1	1.31	18773	24888	n.s.
	39	BC001920.1	actin gamma	Actin, gamma 1	Involved in various types of cell motility, and maintenance of the cytoskeleton.	17q25	1.38	108509	152446	n.s.
	40	NM_001614.2	ACTG1	Actin, gamma 1	Involved in various types of cell motility, and maintenance of the cytoskeleton.	17q25	1.43	113681	166361	n.s.
	41	NM_004368.1	CNN2	Calponin 2	Participates in smooth muscle contractio, cell adhesion and can bind actin.	21q11.1	1.43	4012	5822	n.s.
	42	NM_004994.1	MMP9	Matrix metalloproteinase 9 (gelatinase B, collagenase)	Involved in the breakdown of extracellular matrix in normal physiological processes (embryonic development, reproduction, tissue remodeling), in disease processes (arthritis and metastasis).	20q11.2-q13.1	4.70	3150	16961	n.s.
										
**B) Down-regulated genes (76) in non-melanoma skin cancer.**							**Microarray experiments**			**ANOVA**
								
	**No.**	**Accession No.**	**Symbol**	**Gene**	**Function**	**Localization**	**Change fold N/T**	**Mean of raw signal (normal)**	**Mean of raw signal (AK&SCC)**	**p-value**

**Differentiation**										
	1	NM_004430.1	EGR3	Early growth response 3	Participates in the transcriptional regulation of genes in controling biological rhythm.	8p23-p21	2.22	7833	3782	n.s.
	2	NM_022969.1	FGFR2	Fibroblast growth factor receptor 2	Influences mitogenesis and differentiation.	10q26	2.04	9967	5011	n.s.
	3	BC001971.1	p27, Kip1	Cyclin-dependent kinase inhibitor 1B	Binds to and prevents the activation of cyclin E-CDK2 or cyclin D-CDK4 complexes, and thus controls the cell cycle progression at G1.	12p13.1-p12	1.45	8234	5959	n.s.
	4	**AF309553.1**	REC14	Meiotic recombination protein REC14	Exact function unknown. Thought to be involved in differentiation.	15q24.1	1.96	16148	8395	0.027
	5	**NM_001424.1**	EMP2	Epithelial membrane protein 2	A role in the control of cell proliferation, cell differentiation, and cell death.	16p13.2	2.00	21074	11007	0.026
	6	NM_001983.1	ERCC1*	Excision repair cross-complementing rodent repair deficiency	Repair protein expression is reduced in testis neoplasms.	19q13.2-q13.3	1.79	7222	4073	n.s.
**Apoptosis**										
	7	**NM_016085.1**	**APR-3**^n.s.^	Apoptosis related protein APR-3	Involved in apoptosis, and may also be involved in hematopoietic development and differentiation.	2p23.3	1.85	21640	11863	0.012
	8	AB055804.1	MM-1 beta	MM-1 beta	Assists in the correct folding of other proteins, subunit is thought to repress the transcriptional activity of c-myc.	12q12	1.64	35682	22035	n.s.
	9	NM_015965.1	CGI-39*	CGI-39 protein; cell death-regulatory protein GRIM19	Signal transducer and activator of transcription 3.	19p13.2	1.43	12888	9485	n.s.
**Proliferation**										
	10	BC003689.1	HMG 17	High-mobility group (non-histone chromosomal protein 17)	Nucleosomal binding protein.	1p36.1	1.22	46229	38384	n.s.
	11	**NM_006694.1**	JTB	Jumping translocation breakpoint	Up-regulation in hepatocellular carcinoma (HCC).	1q21	1.39	26313	19283	0.030
	12	**NM_001967.2**	EIF4A2	Eukaryotic translation initiation factor 4A	Protein translation.	3q28	1.92	41342	22341	0.019
	13	NM_005935.1	MLLT2	Myeloidlymphoid or mixed-lineage leukemia	Interaction with SIAH1 and SIAH2 proteins, the cell cycle control exerted by MLL-AF4 may be responsible of resistance to cell-death.	4q21	1.82	14146	8083	n.s.
	14	AF279903.1	EC45	60S ribosomal protein L15	A ribosomal protein that is a component of the 60S subunit.	3p24.1	1.85	60783	36597	n.s.
	15	NM_002948.1	RPL15	Ribosomal protein L15	A ribosomal protein that is a component of the 60S subunit, overexpressed in some esophageal tumors compared to normal matched tissues.	3p24.1	1.79	79109	46326	n.s.
	16	NM_001023.1	RPS20	Ribosomal protein S20	A ribosomal protein that is a component of the 40S subunit.	8q12	1.35	78126	58507	n.s.
	17	NM_000995.1	RPL34	Ribosomal protein L34	Component of the 60S subunit.	4q25	1.85	92356	52954	n.s.
	18	NM_006098.1	GNB2L1	Guanine nucleotide binding protein (G protein)	Regulates G1/S progression by suppressing Src kinase activity.	5q35.3	1.32	73713	57754	n.s.
	19	NM_007104.2	RPL10A	Ribosomal protein L10a	Downregulated in the thymus by cyclosporin-A (CsA), an immunosuppressive drug.	6p21.3-p21.2	1.49	77178	54218	n.s.
	20	BC000734.1	EIF3S6	Eukaryotic translation initiation factor 3	Protein translation.	8q22-q23	1.38	51705	37233	n.s.
	21	NM_003756.1	EIF3S3	Eukaryotic translation initiation factor 3	Protein translation, overexpression of EIF3S3 is associated with breast and prostate cancer.	8q24.11	1.49	38725	25795	n.s.
	22	BC000524.1	RPS6	Ribosomal protein S6	Protein synthesis, cytoplasmic ribosomal protein that is a component of the 40S subunit.	9p21	1.23	99499	83221	n.s.
	23	NM_001417.1	EIF4B	Eukaryotic translation initiation factor 4B	Stimulate the nuclease activity of herpes simplex virus.	12q13.13	1.35	15184	11509	n.s.
	24	NM_000968.1	RPL4	Ribosomal protein L4	Component of the 60S subunit.	15q22	1.30	118180	92600	n.s.
	25	**NM_015920.1**	LOC51065	40S ribosomal protein S27	Similarity with ribosomal protein S27.	15q22.1	1.67	24852	15517	0.043
	26	**NM_001009.1**	RPS5	Ribosomal protein S5	Variable expression of this gene in colorectal cancers compared to adjacent normal tissues, although no correlation between the severity of this disease has been observed.	19q13.4	1.54	57497	38110	0.033
	27	**NM_016091.1**	HSPC025	HSPC025	Interacts with Int-6 and is associated with eukaryotic translation initiation factor 3.	22q	1.45	58905	41562	0.043
	28	L22453.1	TARBP-b	HIV-1 TAR RNA binding protein (RPL3)	Encodes a ribosomal protein that is a component of the 60S subunit; the protein can bind to the HIV-1 TAR mRNA, and the protein probably contributes to tat-mediated transactivation.	22q13	1.47	86865	59742	n.s.
	29	NM_006013.1	RPL10	Ribosomal protein L10	This gene encodes a ribosomal protein that is a component of the 60S subunit.	Xq28	1.27	127604	102770	n.s.
	30	**NM_014315.1**	LCP	host cell factor homolog	Transcriptional coactivator which coordinates the assembly of enhancer complex through direct interactions with viral and cellular trans-activators.	14q21.3-q22.1	1.92	12588	6756	0.003
**Metabolism**										
	31	NM_015958.1	LOC51611	CGI-30 protein	Activities of Dipthine synthase, methyltransferase, and transferase.	1p21.2	1.67	7739	4726	n.s.
	32	**NM_000847**	GSTA3	Glutathione S-transferase A3	Involved in cellular defense against toxic, carcinogenic, and pharmacologically active electrophilic compounds.	6p12.1	5.00	1557	135	0.005
	33	**NM_002633.1**	PGM1	Phosphoglucomutase 1	Regulatory enzyme in cellular glucose utilization and energy homeostasis.	1p31	2.22	22301	10170	0.044
	34	**BC006229.1**	CYC5b	Cytochrome c oxidase subunit Vb	Terminal enzyme of the mitochondrial respiratory chain.	2cen-q13	2.00	17780	9105	0.015
	35	**NM_001862.1**	COX5B	Cytochrome c oxidase subunit Vb	Terminal enzyme of the mitochondrial respiratory chain.	2cen-q13	1.85	26820	14737	0.035
	36	NM_002492.1	NDUFB5	NADH dehydrogenase (ubiquinone) 1 beta subcomplex	A subunit of the multisubunit NADH:ubiquinone oxidoreductase (complex I).	3q27.1	1.67	18898	11821	n.s.
	37	NM_021122.2	FACL2	Fatty-acid-Coenzyme A ligase	Plays a role in lipid biosynthesis and fatty acid degradation.	4q34-q35	2.50	39142	15864	n.s.
	38	BC005270.1	ND4FS4	NADH dehydrogenase (ubiquinone) Fe-S protein 4	Multisubunit enzyme complex of the mitochondrial respiratory chain, plays a vital role in cellular ATP production.	5q11.1	1.85	10920	5956	n.s.
	39	**NM_001867.1**	COX7C	Cytochrome c oxidase subunit VIIc	Mitochondrial respiratory chain, catalyzes the electron transfer from reduced cytochrome c to oxygen.	5q14	2.17	55286	26201	0.013
	40	BC003674.1	NDUFA2	NADH dehydrogenase (ubiquinone) 1 alpha subcomplex	Multisubunit enzyme complex of the mitochondrial respiratory chain, plays a vital role in cellular ATP production.	5q31	1.67	9562	5854	n.s.
	41	NM_014402.1	QP-C	Low molecular mass ubiquinone-binding protein	Activities of oxidoreductase, and ubiquinol-cytochrome c reductase.	5q31.1	1.69	32210	19064	n.s.
	42	BC001917.1	MDH2	Malate dehydrogenase 2, NAD (mitochondrial)	Oxidation of malate to oxaloacetate (citric acid cycle).	7p12.3-q11.2	1.30	24490	19165	n.s.
	43	NM_002489.1	NDUFA4	NADH dehydrogenase (ubiquinone) 1 alpha subcomplex	Multisubunit enzyme complex of the mitochondrial respiratory chain, plays a vital role in cellular ATP production.	7p21.3	1.59	22038	14006	n.s.
	44	NM_005004.1	NDUFB8	NADH dehydrogenase (ubiquinone) 1 beta subcomplex	Multisubunit enzyme complex of the mitochondrial respiratory chain.	10q23.2-q23.33	1.41	25375	18105	n.s.
	45	NM_004074.1	COX8	Cytochrome c oxidase subunit VIII	Terminal enzyme of the respiratory chain.	11q12-q13	1.37	21360	15941	n.s.
	46	NM_004549.1	NDUFC2	NADH dehydrogenase (ubiquinone) 1	Multisubunit enzyme complex of the mitochondrial respiratory chain.	11q13.4	1.59	33921	21642	n.s.
	47	**AF042165**	COX7CP1	Cytochrome c oxidase subunit VIIc	Terminal component of the mitochondrial respiratory chain.	13q14-q21	2.08	40375	19940	0.003
	48	NM_001861.1	COX4	Cytochrome c oxidase subunit IV	Mitochondrial respiratory chain.	16q22-qter	1.25	54196	44561	n.s.
	49	**L47162.1**	FALDH	Human fatty aldehyde dehydrogenase	Detoxification of aldehydes generated by alcohol metabolism and lipid peroxidation.	17p11.2	2.50	16420	6898	0.038
	50	**NM_000382.1**	ALDH3A2	Aldehyde dehydrogenase 3 family	Detoxification of aldehydes generated by alcohol metabolism and lipid peroxidation.	17p11.2	3.33	19513	5867	0.020
	51	NM_021074.1	NDUFV2	NADH dehydrogenase (ubiquinone) flavoprotein 2	Activities of NADH dehydrogenase, electron carrier, and oxidoreductase.	18p11.31-p11.2	1.82	20847	11262	n.s.
	52	M22865.1	CYb5	Human cytochrome b5	Cytochrome c oxidase activity.	18q23	3.57	36175	11654	n.s.
	53	M22976.1	cyt b5	Human cytochrome b5, 3 end	Cytochrome c oxidase activity.	18q23	3.70	22288	6090	n.s.
	54	NM_001914.1	CYB5	Cytochrome b-5	Cytochrome c oxidase activity.	18q23	3.33	31694	10220	n.s.
	55	NM_013387.1	HSPC051	Ubiquinol-cytochrome c reductase complex	Activities of oxidoreductase, and ubiquinol-cytochrome c reductase.	22cen-q12.3	1.43	15592	11200	n.s.
	56	NM_001866.1	COX7B	Cytochrome c oxidase subunit VIIb	Encoded by COX, the terminal component of the mitochondrial respiratory chain, catalyzes the electron transfer from reduced cytochrome c to oxygen.	Xq13.3	1.37	29860	21796	n.s.
	57	**NM_004541.2**	NDUFA1	NADH dehydrogenase (ubiquinone) 1 alpha subcomplex	Codes for an essential component of complex I of the respiratory chain, which transfers electrons from NADH to ubiquinone.	Xq24	2.27	25486	11413	0.026
**Cell communication**										
	58	BC002669.1	NR2	Nuclear receptor subfamily 2	Predicted to encode proteins that are very similar in primary structure to receptors for steroid hormones or thyroid hormone (T3).	19p13.1	1.51	7213	4917	n.s.
	59	NM_004182.1	UXT	Ubiquitously-expressed transcript	Interacts with the N-terminus of the androgen receptor and plays a role in facilitating receptor-induced transcriptional activation.	Xp11.23-p11.22	1.59	11609	7489	n.s.
	60	M92439.1	LeuP	Human leucine-rich protein	Activate expression of MDR1 and MVP (key components of the cytotoxic defense network).	2p21	1.92	6429	3379	n.s.
**Adhesion**										
	61	NM_006893.1	LGTN	Ligatin	A role in neuroplasticity by modulating cell-cell interactions, intracellular adhesion, and protein binding at membrane surfaces.	1q31-q32	1.70	9444	5566	n.s.
	62	M80927.1	CHI3L1**	Human glycoprotein -39	Extracellular matrix structural constituent, sugar binding, and hydrolase activity.	1q32.1	7.69	31265	5550	n.s.
	63	**NM_015717.1**	LANGERIN	Langerhans cell specific c-type lectin	Expressed only in Langerhans cells which are immature dendritic cells of the epidermis and mucosa.	2p13	4.00	4586	1323	0.023
	64	NM_007234.2	DCTN3	Dynactin 3 (p22)	Involved in a diverse array of cellular functions, including ER-to-Golgi transport, the centripetal movement of lysosomes and endosomes, spindle formation, cytokinesis, chromosome movement, nuclear positioning, and axonogenesis.	9p13	1.45	13690	9694	n.s.
	65	NM_018663.1	LOC55895	Peroxisomal membrane protein	Peroxisome organization and biogenesis (assembly and arrangement of peroxisome).	12q24.33	3.45	6928	2170	n.s.
**Detoxification**										
	66	NM_001512.1	GSTA4	Glutathione S-transferase A4	Involved in cellular defense against toxic, carcinogenic, and pharmacologically active electrophilic compounds.	6p12.1	1.41	25838	18009	n.s.
	67	**NM_004528.1**	MGST3	Microsomal glutathione S-transferase 3	Involved in the production of leukotrienes and prostaglandin E, important mediators of inflammation.	1q23	1.96	22933	12007	0.023
	68	NM_002413.1	MGST2	Microsomal glutathione S-transferase 2	Catalyzes the conjugation of leukotriene A4 and reduced glutathione to produce leukotriene C4.	4q28.3	1.82	10888	6065	n.s.
	69	NM_015917.1	LOC51064	Glutathione S-transferase subunit 13 homolog	Activities of glutathione transferase, protein disulfide oxidoreductase, and transferase.	7	1.75	9422	5458	n.s.
	70	NM_001752.1	CAT	Catalase	Abnormal expression of catalase in the eutopic and ectopic endometrium strongly suggests pathologic involvement of free radicals in endometriosis and adenomyosis.	11p13	1.61	27669	17923	n.s.
	71	L19185	**NKEFB**^n.s.^	Human natural killer cell enhancing factor	Reduce hydrogen peroxide and alkyl hydroperoxides.	19p13.2	2.50	39723	17286	n.s.
**unknown**										
	72	**NM_016098.1**	LOC51660	HSPC040 protein	Unknown Function.	6q27	3.33	15481	4682	0.038
	73	**AL356115**	KIAA1128	KIAA1128 protein	Unknown Function.	10q23.2	1.38	126962	93488	0.048
	74	AK022248	FLJ12186	FLJ12186	Unknown Function.	14q22.3	1.38	15261	11116	n.s.
	75	NM_004868.1	GPSN2	Glycoprotein, synaptic 2	Unknown Function.	19p13.12	1.69	19830	11888	n.s.
	76	**AF151056.1**	HSPC222	HSPC222	Unknown Function.	unknown	1.59	40513	26566	0.030

The accession number, the symbol, the description of the genes, their function, their chromosome localization, the change fold, the raw signal (mean value), and the p values of the ANOVA analysis are shown. The accession numbers in bold represent the 42 genes identified by PAM and ANOVA (p < 0.05), which were significantly differentially expressed. The symbols marked in bold represent the genes verified by quantitative real-time RT-PCR.

A) Up-regulated genes (42) in non-melanoma skin cancer.

B) Down-regulated genes (76) in non – melanoma skin cancer.

* significant expression difference verified by quantitative real-time RT-PCR.

** positive with two different affymetrix numbers (209395_at and 209396_s_at).

No., numbers; T, Tumor (AK and SCC), N, normal skin; AK, actinic keratosis; SCC, squamous cell carcinoma; n.s., not significant.

**Figure 1:**
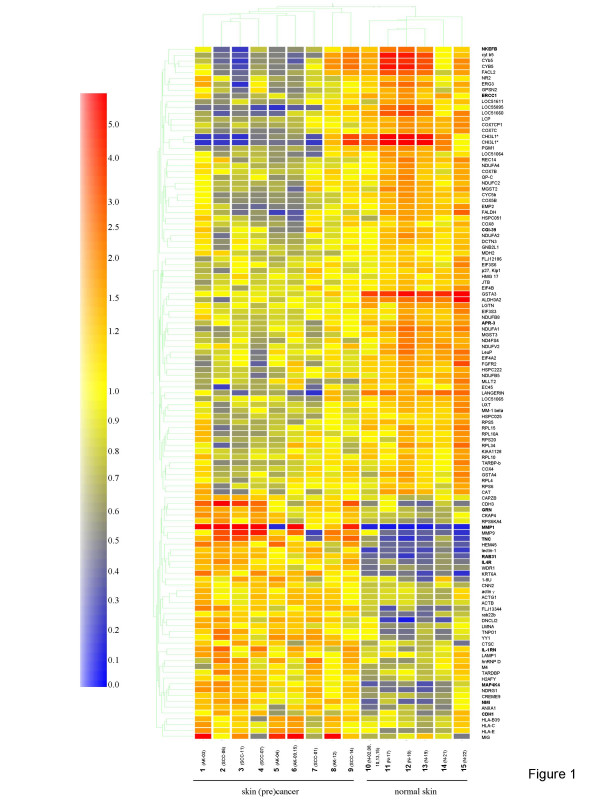
**Cluster map analysis of 118 genes identified by PAM of 15 different specimens resulting in two classes (9 neoplastic skin lesions and 6 normal skin)**. Prediction Analysis of Microarrays (PAM) using nearest shrunken centroids was performed with 22,283 genes, which were present on the microarray platform (Affymetrix) to identify genes best characterizing normal skin, actinic keratosis (AK), and squamous cell carcinoma (SCC). Hierarchical clustering was performed with 118 genes identified by PAM (CHI3L1 was detected with two independent Affymetrix probes and are included twice, marked with an asterisk). Thirteen genes verified by quantitative real-time RT-PCR are marked in bold. Each color patch represents the normalized expression level of one gene in each group, with a continuum of expression levels from dark blue (lowest) to dark red (highest). The minimal set of informative genes is given by HUGO Gene Nomenclature Committee (HGNC) symbols. Group (1–9) are non-melanoma skin cancer AK (1, 5, 6, and 8), and SCC (2–4, 7, and 9) showing different expression levels compared with six cases of normal skin (10–15). Numbers 6 and 10 were specimens with pooled RNAs.

Hierarchical clustering was performed with the identified 118 genes based on similarities of expression levels independently of the assigned class (normal skin, AK or SCC). Gene trees display gene similarities as a dendrogram, a tree-like structure made up of branches. This nested structure forces all genes to be related to a certain level, with larger branches representing the more distantly related genes (Figure 1). The gene *CHI3L1 *was detected with two different affymetrix numbers and are included twice in the cluster map (Figure 1). The "Condition Tree" groups samples together based on similar expression profiles by standard correlation with the GeneSpring software 6.1 resulting in two classes. The specimens with the pooled RNA (normal and AK) grouped together with the non-pooled RNA in class 1 (normal skin) and class 2 (AK and SCC) (Figure 1). In class 1, all 6 normal skin specimens grouped together, and class 2 consisted of 4 AK and 5 SCC. Thus, statistical differences in the expression levels of such genes were not detected in carcinoma *in situ *(AK) *vs*. invasive cancer (SCC). Furthermore, the pooled normal skin specimens from 5 immunosuppressed patients grouped together with 5 non-pooled normal skin specimens from immunocompetent patients (Figure 1). Thus, the expression levels of the selected genes were independent of systemic immunosuppression.

ANOVA analysis identified 364 genes including 7 EST, which were significantly differentially expressed between normal skin, AK, and cutaneous SCC (p < 0.05). Using p < 0.15 the gene list contained 2,197 genes including 42 EST. The overall agreement rate of the identified genes by ANOVA using p < 0.05 or p < 0.15 and PAM was 36% (42 of 118) or 78% (92 of 118), respectively. To identify potentially dysregulated genes between AK and SCC, we have performed ANOVA analysis in these two groups (p < 0.05), and no gene was significantly differentially expressed in AK compared with SCC. For further analysis we have used the 118 genes that have been identified by PAM and the 42 significantly differentially expressed genes identified by both methods are highlighted in Table 2.

A higher expression level in skin tumors *vs*. normal skin was observed for 42 genes. Of these genes 19 (45%) were involved in adhesion, 13 (31%) in cell communication, 6 (14%) in metabolism, two in differentiation (5%), and one each in apoptosis (2%) and in proliferation (2%). In contrast, a lower expression rate was observed for 76 genes and 27 (36%) were involved in metabolism, 21 (28%) in proliferation, 6 (8%) each in differentiation, and detoxification, 5 (7%) in adhesion, 3 (4%) each in apoptosis, and cell communication, and 5 (7%) were of unknown functions.

### Verification of selected genes by quantitative real-time RT-PCR

**Figure 2:**
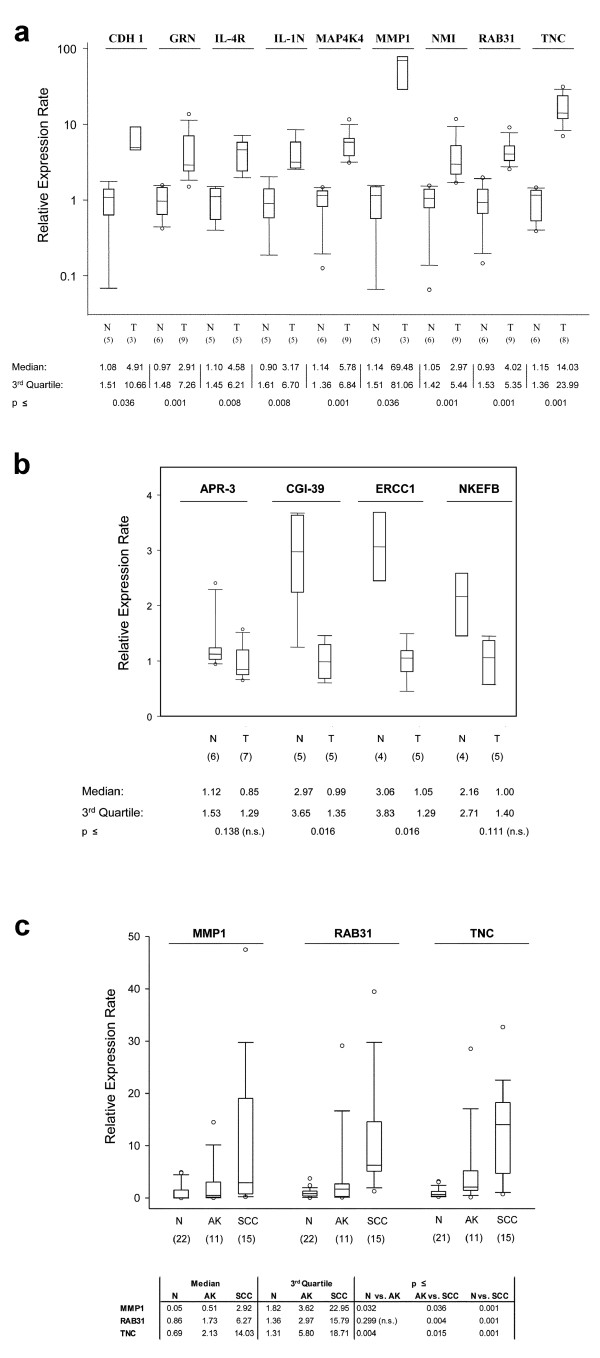
**Verification of 13 genes by quantitative real-time RT-PCR**. Expression levels were based on the amount of the target RNA relatively to the endogenous control gene RPS9 in order to normalize the amount and quality of total RNA. Relative expression levels of a) 9 up-regulated genes, b) 4 down-regulated genes, and c) 3 up-regulated genes in epithelial skin cancer *vs*. normal skin. c) Three genes were re-analyzed with an increased number of different specimens of immunocompetent and immunosuppressed patients (see Methods section 'Patients'). Statistical analysis was performed with the U-Test by Wilcoxon, Mann, and Whitney. N, normal skin; T, AK and cutaneous SCC; n.s., not significant; AK, actinic keratosis; SCC, squamous cell carcinoma.

To verify the different expression levels of mRNA measured by microarray technology, we selected 13 up- or down-regulated genes with low through high change folds from the list of differentially expressed genes. These included 9 genes with a higher (change folds by microarray analysis 1.31 – >10) and 4 with a lower expression level (change folds by microarray analysis 1.43 – 2.50) in neoplastic skin lesions *vs*. normal skin (Table 2). Gene specific intron-flanking primers were designed for 9 up-regulated genes (*RAB31*, *MAP4K4*, *IL-1RN*, *NMI*, *IL-4R*, *GRN*, *TNC*, *MMP1*, and *CDH1*) and 4 down-regulated genes (*ERCC1, APR-3, CGI-39*, and *NKEFB*) (Table 3). Unspecific PCR products were not obtained for all genes shown by electrophoresis of the PCR amplicons in agarose gels. Gene-specificity of all 13 genes was confirmed by sequencing of the PCR product of each gene. The results of the real-time RT-PCR were consistent with the microarray data (Figure 2). All genes showed the predicted expression level either higher or lower in normal skin *vs*. skin cancer. The real-time RT-PCR data confirmed significant expression differences in 11 of 13 genes (85%) (p < 0.05) (Figure 2). *APR-3 *and *NKEFB *showed the predicted lower expression level in skin tumors *vs*. normal skin (median values 0.85 *vs*. 1.12, p = 0.14, median values 1.0 *vs*. 2.16, p = 0.11). Furthermore, we reanalyzed 3 of 13 genes with an increased number of specimens of immunocompetent and immunosuppressed patients with normal skin (n ≥ 21), AK (n = 11), and SCC (n = 15). The change folds by microarray analysis of the three up-regulated genes *MMP1, TNC*, and *RAB31 *were >10, 4.6, and 3.3, respectively. The median expression rate by real-time RT-PCR in normal skin (N) *vs*. AK *vs*. SCC of *MMP1 *was 0.1 *vs*. 0.5 *vs*. 2.9 (N *vs*. SCC, p ≤ 0.001), of *TNC *0.7 *vs*. 2.1 *vs*. 14.0 (N *vs*. SCC, p ≤ 0.001), and of *RAB31 *0.9 *vs*. 1.7 *vs*. 6.3 ((N *vs*. SCC, p ≤ 0.001), respectively.

**Table 3:** Forward and reverse primer sequences for quantitative real-time RT-PCR to verify differentially expressed genes.

Gene	Forward (5'-3')	Reverse (5'-3')
APR-3	GGT TCT GAT TTC GTC CCT GA	CAG CAT TAG CTC TCG TGT CG
CDH1	TGA AGG TGA CAG AGC CTC TGG AT	TGG GTG AAT TCG GGC TTG TT
CGI-39	CGT CAA AGG TGA AGC AGG AC	ATT ATG CTC CAG TGC CCG TA
ERCC1	GGG AAT TTG GCG ACG TAA TTC	GCG GAG GCT GAG GAA CAG
GRN	CAG TGG GAA GTA TGG CTG CT	TTA GTG AGG AGG TCC GTG GT

IL-1RN	GGA AGA TGT GCC TGT CCT GT	CGC TTG TCC TGC TTT CTG TT
IL4R	CAC CTG CCT CTG TCT CAC TGA A	GGC CGC CCA AGT CAT TC
MAP4K4	CTG GTC ACT TGG ATG GTG TG	AGA CCG AAC AGA GGC AAA GA
MMP1	CTT GCA CTG AGA AAG AAG ACA AAG G	ACA CCC CAG AAC AGC AGC A
NKEFB	ACC CAG GAA AGG GCA GAC	TTC TAG GTG GAG GCA TTG AGA

NMI	GAC ACA CTG CGT GAA GAT CAA	TCC AAT CTC CAC AAA CGT GA
RAB31	TGA CCA CAA CAT CAG CCC TA	AAT GAA ACC GTT CCT GAC CA
TNC	ACT GTG GAC GGA ACC AAG AC	TGT GGT GAA TGA CCC TGA GA
		
RPS9	ATC CGC CAG CGC CAT A	TCA ATG TGC TTC TGG GAA TCC

## Discussion

We have examined the expression levels of 22,283 genes in human biopsies of normal skin and cutaneous squamous cell carcinoma (AK, and SCC) by microarray technology. One hundred and eighteen genes were differentially expressed in normal skin *vs*. skin cancer and fulfilled the criteria used for PAM based cluster map analysis.

Expression profiling using oligonucleotide microarrays is a useful tool to identify tumors, to distinguish different tumor entities, and to differentiate between progressing and non-progressing neoplastic lesions [7,15-18]. In this study, we used mRNA from skin biopsies without microdissection resulting in high RNA amounts and subsequently no amplification of the RNA transcripts were required. On the other hand, we cannot avoid a mixture of dysplastic and non-dysplastic cells in our specimens. A mixture of normal epithelial cells and tumor cells are most likely present in cancerous lesions (AK) but are unlikely in normal skin specimens. If the tumor specimen contained normal and dysplastic cells, an increased gene expression in cancer *vs*. normal skin or vice versa was not detected by microarray analysis. Thus, the number of differentially expressed genes detected in our study represent a subset of all differentially expressed genes in skin cancer and genes showing only low differences are most likely to be unidentified.

The number of differentially expressed genes in normal tissue *vs*. colon cancer and breast cancer was 548 and 700, respectively [7,8]. In our study, we detected 118 genes excluding EST best characterizing normal skin and epithelial skin cancer. These genes represent approximately 20% of all genes expected to be differentially expressed in skin cancer. So far, only one study examined the expression profile in human biopsies of NMSC and skin cancer cell lines by microarray analyzing approximately 7,400 genes [14]. Although there was only a minimal overlap between human tissue and cell lines, five genes were differentially expressed both *in vivo *and *in vitro*, namely *fibronectin 1, annexin A5, glyceraldehyde 3-phosphate dehydrogenase, zink-finger protein 254*, and *huntingtin-associated protein interacting protein*. Of these genes the calcium and phospholipid-binding protein a*nnexin 5 *was over-expressed (ratio 2.1) and *annexin 1 *showed a slightly over-expression in cutaneous SCC. In our study using another approach *annexin 1 *was over-expressed in AK and cutaneous SCC (change fold 1.74) showing that 1 of 5 genes (20%) differentially expressed in the study of Dooley and colleagues [14] could be confirmed. *Lamin A and C *showed a significant higher expression in AK and SCC compared with normal skin analyzed by immunohistochemistry [19], and these genes were also up-regulated in our study. Furthermore, enzymes of the mitochondrial chain namely *cytochrome c oxidase*, *cytochrome b*, and *NADH dehydrogenase *were the majority of down-regulated genes. In prostatic intra-epithelial neoplasia, a high mutation rate of *NADH *subunits of the respiratory chain complex I was observed similar to lung and head and neck cancer [20]. Delsite and colleagues [21] examined breast cancer cell lines and suggested that a lack of mitochondrial genes leads to increased oxidative stress, reduced DNA repair, and genetic instability. Furthermore, mitochondrial dysfunction leads to an increased production of reactive oxygene species (ROS), inhibition of apoptosis, activation of oncogenes, and inactivation of tumor suppressor genes, and thus is involved during carcinogenesis [22,23]. In our study, the majority of these enzymes was down-regulated in NMSC, indicating that mitochondrial dysfunction is possible associated with cutaneous SCC.

The reliability of the identified 118 genes by microarray technology was verified and confirmed by real-time RT-PCR analyzing 13 genes, and the following discussion is based on these genes.

### 9 genes up-regulated in NMSC (*CDH1*, *MAP4K4*, *IL-1RN*, *IL-4R*, *NMI*, *GRN*, *RAB31*, *TNC*, and *MMP1*)

*CDH1 *(*E-Cadherin*) is a representative of the classic cadherin family and a calcium-dependent cell-cell adhesion glycoprotein, mutations are correlated with a variety of cancers and loss of function may lead to cancer progression and metastasis [24]. In our study we observed an over-expression of *CDH1 *in skin cancer *vs*. normal skin, although a lower expression rate was expected. This may be due to a wrongly identified gene, a loss of function due to mutations, a post-transcriptional regulation of this gene or another mechanism in skin cancer *vs*. other cancers.

*MAP4K4 *is a representative of the serine/threonine protein kinase family activating *MAPK8/c-Jun N-terminal kinase (JNK*) [25]. *JNK *signal transduction pathway participates in the proliferation, differentiation, and apoptosis of osteoblasts and is functionally operative in the malignant transformation of osteoblasts and the subsequent development and progression of human osteosarcomas [26]. *IL-1RN *(*interleukin 1 receptor antagonist*), *IL-4R *(*interleukin 4 receptor*), *NMI *(*N-Myc *and *Stat *interactor), and *GRN *(*granulin*) are genes involved in cell communications. *IL-1RN *is a representative of the interleukin 1 cytokine family inhibiting the activities of *IL-1A *and *IL-1B*, and modulates a variety of interleukin 1 related immune and inflammatory responses [27]. The homozygous genotype IL-1RN*2/2 of the *IL-RN *gene was strongly associated with early-stage gastric cancer [28]. IL-4R develop allergic reactions, modulate the function of monocytes and macrophages and has been shown over-expressed in a variety of human cancer cells *in vitro *and *in vivo *like melanoma, breast, ovarian, renal, and head and neck [29]. *NMI *interacts with the oncogenes *C-myc *and *N-myc *and other transcription factors containing a ZIP, HLH, or HLH-Zip motif first isolated and characterized by Bao and Zervos [30]. In addition, NMI interacts with all Stats except of Stat2 and augments Stat-mediated transcription in response to cytokines IL-2 and IFN-gamma [31]. A novel pathogenic mechanism of the transcription factor complex NMI, BRCA1 and c-Myc is the activation of telomerase, which is a key enzyme in carcinogenesis [32]. The growth factor *GRN *stimulates progression and metastasis of breast cancer and is involved in a variety of cancers such as clear cell renal carcinoma, invasive ovarian carcinoma and glioblastoma [33]. In our study all 5 genes showing different functions in tumorigenesis were also over-expressed in skin cancer.

*Rab31 *represent a family of monomeric GTP-binding proteins and belongs to the Ras family [34,35]. *Ras *is a proto-oncogene, which is evolutionary conserved and is involved in various cancers [36], but the precise role of *Ras*, especially *Ha-ras *in NMSC is unknown. *TNC *is an extracellular matrix protein with anti-adhesive effects, and involved in tissue interactions during fetal development and oncogenesis. *TNC *was associated with breast and lung cancer [37] and was over-expressed in vulvar intraepithelial neoplasia. *Matrix metalloproteinases *(*MMP*) are involved in extracellular matrix degradation and cancer invasion [38,39]. Tsukifuji and colleagues [40] reported an over-expression of *MMP-1*, *MMP-2*, and *MMP-3 *in skin cancer (16 Ak, 6 AK with SCC, and 15 SCC). We detected an increased expression rate of *MMP-1 *and *MMP-9 *in skin cancer and both genes showed the highest expression rate in AK/SCC indicated by the change-folds of *MMP-1 *(<10), and *MMP-9 *(4.70) by microarray analysis. All three genes are considered to be involved in a variety of cancers, they were over-expressed in our study and may play a role in the cancerogenesis of NMSC, and thus are interesting candidates for further studies.

### 4 genes down-regulated in NMSC (*ERCC1*, *APR-3*, *CGI-39*, and *NKEFB*)

*ERCC1 *has a high homology with the yeast excision repair protein *RAD10 *[41], is reduced in testis neoplasms [42] and ovarian cancer cell lines [43]. *APR-3 *is considered to be involved in apoptosis and was identified using subtractive hybridization strategy in order to clone apoptosis-related genes [44]. *NKEFB *encodes a representative of the peroxiredoxin (Prx) family of antioxidant enzymes and may play a role in cancer development [45]. Prx II was strongly expressed in mature endothelial cells of benign vascular tumors, whereas it was weakly or not expressed in immature endothelial cells in malignant tumors of Kaposi's sarcoma and angiosarcoma [46]. In our study these genes were also down-regulated in skin cancer, and thus were consistent with the expected expression level observed in other carcinoma.

## Conclusion

In conclusion, we identified 42 genes up-regulated and 76 genes down-regulated in cutaneous squamous cell carcinoma (AK and SCC) *vs*. normal skin, which represent approximately 20% of the genes differentially expressed in skin cancer. The majority of genes which known functions in other cancers was consistent with our results of differentially expressed genes in NMSC. These 118 genes either individually or more likely together or a subset of these genes may prove useful for diagnostic approaches.

## Methods

### Patients

Biopsies were obtained from 5 organ-transplanted (TX) recipients (3 kidney, 1 heart, and 1 liver, 58–73 years, median age 66 years) each normal skin, AK, and SCC. The time since transplantation ranged from 2 through 23 years (median 11 years), and no rejection was observed. All patients had multiple NMSC, such as AK, SCC and/or basal cell carcinoma, and lesions were mainly located on sun-exposed areas. The specimens from TX recipients of 5 normal skin and 2 AK specimens were pooled due to the low RNA amount of the individual specimens that was not sufficient for further microarray analyses. Furthermore, we have included 5 normal skin specimens from age-matched non-immunosuppressed individuals (17–74 years, median age 61 years). Thus, we have analyzed 6 normal skin, 4 AK, and 5 SCC specimens by microarray technology (Table 1). All clinical specimens were collected under standardized conditions by the same clinician (TF). From each organ-transplanted patient, punch biopsies (diameter 4 mm) of normal tissue, AK, and SCC were collected. Half of the tissue was transferred to liquid nitrogen within 2 minutes of resection and stored at -70°C until RNA isolation was performed. The other half of each biopsy was fixed in formalin, embedded in paraffin and sections were stained with hematoxylin and eosin for histological evaluation. All clinical diagnoses, normal skin, AK, and SCC were confirmed by histology.

The same 15 RNA specimens (or representative subsets) were used for quantitative real-time reverse transcription (RT)-PCR of 13 selected genes for verification (Figure 2). Furthermore, 3 of these 13 genes *MMP1*, *RAB31*, and *TNC *were additionally examined by real-time RT-PCR with different specimens. For this analysis we used specimens of 22 normal skin (50–79 years, median age 63 years, including one immunosuppressed patient), of 11 AK (55–83 years, median age 63 years, including 7 immunosuppressed patients), and of 15 SCC patients (46–84 years, median age 61 years, including 7 immunosuppressed patients). The RNA amount of one normal skin specimen was not sufficient for TNC analysis, reducing the number of samples from 22 to 21 in this analysis. The study was approved by the local ethics committee at the Charite, University Hospital, Berlin, Germany (number Si. 248).

### RNA isolation and microarray hybridization

Total RNA was isolated using a modified RNeasy Micro Kit protocol (Qiagen, Hilden, Germany). The modification included the homogenization of the frozen tissue in 300 μl of buffer RLT (Qiagen) with 20 ng Glycogen (Roche, Mannheim, Germany) using a rotar-stator homogenizer "Ultra Turrax T25" (Janke & Kunkel, Staufen, Germany). The homogenized tissue was digested with 10 μl Proteinase K (10 mg ml^-1^) (Roth, Karlsruhe, Germany) at 55°C for 15 min. Subsequently the sample was digested with DNase I (Invitrogen, Karlsruhe, Germany). Quantification of isolated RNA was performed using UV-spectroscopy and the quality was determined both by A_260_/A_280 _ratio and Agilent bioanalyzer (Agilent Technologies, Santa Clara, CA, USA). Five microgram total RNA was used for cDNA synthesis with 5 pmol μl^-1 ^T7-oligo(dT)_24 _primer and was performed at 43°C for 90 minutes with the "Superscript First-Strand Synthesis-System" for RT-PCR (Invitrogen). Second-strand synthesis was performed with complete cDNA. The cDNA solution was incubated at 16°C for 2 hours followed by an incubation step for 20 min with 6 U T4-DNA polymerase at 16°C and the reaction was stopped using 10 μl of 0.5 M EDTA. The double stranded cDNA was purified by phenol/chloroform, ethanol precipitated and the pellet was resuspended in 12 μl of DEPC water. Labeled cRNA was generated from the cDNA sample by an *in vitro *transcription reaction that was supplemented with biotin-11-CTP and biotin-16-UTP (Enzo Diagnostics, Farmingdale, NY, USA) according to the manufacturer. The cRNA was quantified by A_260_, and the quality was determined using the labchip bioanalyzer (Agilent). Only cRNA specimens with a high quality were selected for further analyses. Fragmented cRNA (15 μg) was used to prepare 300 μl hybridization cocktail (100 mM MES, 1 M NaCl, 20 mM EDTA, 0.01% Tween-20) containing 0.1 mg ml^-1 ^of herring sperm DNA, and 0.5 mg ml^-1 ^acetylated bovine serum albumine. Control cRNA was used in order to compare hybridization efficiencies between arrays and to standardize the quantification of measured transcript levels and was included as component of the 'Eukaryotic Hybridization Control kit' (Affymetrix, Santa Clara, CA, USA). The cocktails were heated to 95°C for 5 minutes, equilibrated at 45°C for 5 minutes, and clarified by centrifugation. The cocktail was hybridized to HG U133A arrays (Affymetrix) at 45°C for 16 hours. The arrays were washed and stained with a streptavidin-conjugated fluor using the GeneChip fluidics station protocol EukGE-WS2 (Affymetrix) according to the manufacturer's instructions. Arrays were scanned with an argon-ion laser confocal scanner (Hewlett-Packard, Santa Clara, CA) with detection at 570 nm. Data were extracted using Microarray Suite version 5.0 (Affymetrix) and linearly scaled to achieve an average intensity of 2,500 per gene. Text files were exported to determine the intensity of each interrogating oligonucleotide perfect match probe cells or mismatch probe cells. In addition, the ratios of 5'- and 3'-ends of mRNA were analyzed of six randomly selected specimens (two of each group) using microarray test-chips (Test3 Array) containing 24 human housekeeping/maintenance genes (Affymetrix) and RNA degradation was not observed.

### Bioinformatic analysis

The Data Mining Tool 3.0 (Affymetrix) and GeneSpring software package 6.1 (Silicon Genetics, Redwood City, CA, USA) were used for different replicates and statistical analyses were performed in order to compare between cancer stages. For each hybridization, the intensities were normalized in three steps, (1) data transformation, (2) per chip, and (3) per gene. (1) All values below 300 were set to 300, (2) each chip was normalized to the 50^th ^percentile of the measurements, and (3) the median of the intensities of each probe sets representing one gene of all 15 microarray experiments was taken. The normalized values were used for further analyses.

All processings from raw data, normalized raw data and p-detection values of the microarray experiments to the final tables and figures and a description of the method are provided as supplemental material with the series number GSE2503 [47]. Thus, the entire process of analysis is completely transparent and the description of the methodology used is according to the MIAME standard (minimum information about a microarray experiment).

We used PAM for classification of tumors and identifications of genes that were significantly different expressed between three groups. PAM is a statistical technique for class prediction from gene expression data using nearest shrunken centroids. The technique has advantages in accuracy, especially when more than two classes are considered to be examined [48,49] as it is required for this study. PAM ranks genes using a panelized t-statistic and uses soft-thresholding to identify a gene set for classification. Data analysis was performed with 22,283 genes of all 15 specimens depending on their class (normal skin, AK, or SCC). The number of genes used was controlled by a thresholding parameter, which was determined with a 10-fold cross-validation. We used the imputation engine method with the k-nearest neighbor (n = 10), and the threshold 3.5 was chosen to minimize the overall error rate. This cross validation also allows a judgment of the classification quality. For the detailed mathematic procedure, see Tibshirani et al. [48].

In addition, we have independently applied the ANOVA model using two different p-values (p < 0.05 and p < 0.15) to identify dysregulated genes between three groups (normal skin, AK, and SCC) and two groups (AK and SCC) to focus on differences between these two groups. Multiple testing corrections were performed by the false discovery rate of Benjamini and Hochberg for all analyses.

Hierarchical clustering was performed with the genes best characterizing normal skin and NMSC identified by PAM. Genes of all 15 specimens with different expression profiles were grouped by standard correlation with GeneSpring software package 6.1. Hierarchical clustering of the genes was based on similarities of expression levels.

### Quantitative real-time RT-PCR

Real-time RT-PCR with the LightCycler system (Roche) was used as an independent method to validate the microarray expression data and to assess quantitative gene expression. Thirteen genes were selected including 9 up-regulated and 4 down-regulated genes in NMSC with low, moderate, and high change folds. In addition, 3 of the 13 genes (*MMP1, RAB31*, and *TNC*) were verified with an increased number of different specimens of immunosuppressed and immunocompetent patients (22 normal skin, 11 AK, and 15 SCC) (see Methods section 'Patients'). RT was performed with the "Superscript First-Strand Synthesis-System" (Invitrogen) using oligo-dT as described by the manufacturer. The concentration of cDNA was quantified with "OliGreen ssDNA Quantitation Kit" (Molecular Probes, Leiden, Netherlands). Specific PCR primers for the target genes were designed using the Primer3 software program [50], and synthesized by Metabion (Planegg-Martinsried, Germany). The primers of each gene were located in different exons to exclude DNA contamination. Amplification mix (20 μl) contained 20 ng of cDNA, 500 nM of each primer, 2 μl LightCycler FastStart Reaction Mix Syber Green I (Roche), 3 mM MgCl_2 _and sterile double distilled water. The concentration of MgCl_2 _varied depending on each specific primer pair between 3–5 mM. PCR reaction was initiated with 10 min denaturation at 95°C followed by 40 cycles (95°C for 10 sec, 60°C for 5 sec, and 72°C for 10 sec). Fluorescence detection was performed immediately at the end of each annealing step and the purity of each amplification product was confirmed by generating melting curves. All specific RT-PCR products of target genes were purified by gel extraction and confirmed by sequencing with gene specific primers (Table 3) using the DNA sequencing kit and the ABI PRISM 310 Genetic Analyzer (Applied Biosystems, Foster City, USA). A negative control without reverse transcriptase was included in each PCR experiment. The expression of RPS9 was used to control equal RNA loading and to normalize relative expression data for all other genes analyzed. The copy ratio of each analyzed cDNA was determined as the mean of two experiments. The U-Test of Wilcoxon, Mann, and Whitney was applied for estimation of differentially expressed transcripts identified by real-time RT-PCR. A p-value < 0.05 was considered significant for alpha.

## Abbreviations

AK, actinic keratosis; EST, expressed sequence tag; NMSC, non-melanoma skin cancer; RT, reverse transcription; SCC, squamous cell carcinoma; TX, organ transplant; UV, ultraviolett radiation; PAM, predicition analysis of microarrays

## Competing interests

The author(s) declare that they have no competing interests.

## Authors' contributions

All authors contributed equally to this manuscript.
